# Posteriorly positioned femoral grafts decrease long-term failure in anterior cruciate ligament reconstruction, femoral and tibial graft positions did not affect long-term reported outcome

**DOI:** 10.1007/s00167-022-06871-1

**Published:** 2022-02-02

**Authors:** Tim T. C. R. de Mees, Max Reijman, Jan Hendrik Waarsing, Duncan E. Meuffels

**Affiliations:** Department of Orthopaedic Surgery, Erasmus MC, University Medical Center Rotterdam, Dr. Molewaterplein 40, 3015 GD Rotterdam, The Netherlands

**Keywords:** Anterior cruciate ligament reconstruction, Trunnel positioning, Graft placement, Long-term outcome, Graft failure, IKDC

## Abstract

**Purpose:**

To investigate the effect that femoral and tibial tunnel positions have on long-term reported and clinical outcome and to identify a safe zone based on favourable outcome.

**Methods:**

Seventy-eight patients from a previous randomised controlled trial were included and were followed with a mean follow-up of 11.4 years. All patients had primary trans-tibial anterior cruciate ligament reconstruction performed. The femoral and tibial tunnel positions were visualised and translated in percentages with three-dimensional computed tomography post-operatively. There were 3 separate outcome variables: patient-reported outcome measured with the IKDC Subjective Knee Form, overall failure, and radiographic osteoarthritis. The correlation between tunnel aperture positions and outcome was determined with multivariate regression. The area with best outcome was defined as the safe zone and was determined with Youden’s index in conjunction with receiver operating characteristics.

**Results:**

No significant relationship was found between tunnel aperture positions and IKDC Subjective Knee Form at 10-year follow-up. The posterior-to-anterior femoral tunnel aperture position parallel to Blumensaat line showed a significant relationship (*p* = 0.03) to overall failure at 10-year follow-up. The mean posterior-to-anterior tunnel position of the group that did not fail was 37.7% compared to 44.1% in the overall failure group. Femoral tunnel apertures placed further anteriorly had more overall failures at long-term. The cut-off point lies at 35.0% from posterior-to-anterior parallel to Blumensaat. Of the 16 overall failures, 15 (93.8%) were placed further anteriorly than the cut-off point. No significant relationship was found between tunnel aperture positions and radiographic osteoarthritis.

**Conclusion:**

Femoral and tibial tunnel positions were not associated with long-term patient-reported outcome and radiographic osteoarthritis. Long-term overall failure was more frequently seen in patients with a more anteriorly placed femoral tunnel. This study identified a safe zone located at the most posterior 35% of the femoral condyle parallel to Blumensaat. This knowledge offers guidance to surgeons to operate more precisely and accurately and reconstruct a long-lasting graft.

**Level of evidence:**

Level III.

## Introduction

Achieving anatomical resemblance in graft positioning is an important goal in anterior cruciate ligament (ACL) reconstruction, but is also a goal of great complexity. The surgeon has to consider numerous factors whilst operating within the anatomic boundaries of the femoral intercondylar notch and tibial eminences. Of these factors, tunnel positioning is most essential to achieve an anatomic graft position and an excellent ACL reconstruction [2, 3, 9, 20, 21, 23, 24, 28, 36, 40, 41, 43, 46, 51, 57].

Improper graft position leads to decreased patient-reported outcome, increased knee laxity and increased failure rate [3, 20, 21, 28, 36, 43, 46]. The anatomical explanations for this are graft impingement in the roof of the intercondylar notch, excessive graft forces, graft laxity, and tunnel widening [2, 17, 24, 28, 32, 36, 55]. Several studies found an association between tunnel positions and outcome, especially at short term [2, 3, 9, 21, 24, 36, 43]. Principally, the tunnel positions should be anatomically oriented to create optimal stability. The femoral tunnel aperture position resembles the anatomy best if it is placed posteriorly and around middle-height in the lateral condyle [5, 31, 36, 39, 55, 60]. The tibial tunnel aperture position resembles the ACL best when placed between the eminences and slightly more anteriorly than the midline [4, 8, 15, 28, 31, 32, 39, 47].

However, between all these associations, there is no consensus yet about which exact tunnel positions resemble the anatomy best. The most precise visualisation of these tunnel aperture positions can be obtained with three-dimensional computed tomography (3D-CT). This imaging technique provides a 100% visualisation with excellent intra- and inter-observer reliability [25, 30].

The long-term functioning of a graft, which is measured with different long-term outcome parameters, is essential to retain patient satisfaction and high activity levels [13, 44]. Long-term outcome has different dimensions and exists of patient-reported outcome, clinical outcome (such as knee laxity) and graft failure and revision (due to graft ruptures). There is a limited number of studies that linked this long-term outcome to radiographically assessed tunnel positions [15, 41, 50]. However, the relation between long-term outcome and precisely visualised femoral and tibial tunnel aperture positions with 3D-CT has not been studied yet. The purpose of our study was to investigate the effect that femoral and tibial tunnel positions have on long-term reported and clinical outcome and to identify a safe zone based on favourable outcome. The hypothesis is that femoral and tibial tunnel apertures placed closer to the anatomic ACL positions show more favourable long-term outcome compared to non-anatomically placed tunnel positions [5, 7, 11, 47]. Knowledge about the relation between exact tunnel positions and long-term outcome is clinically relevant for surgeons to operate more precise and accurate and reconstruct a long-lasting graft.

## Materials and methods

This is a retrospective cohort study with a prospective follow-up examination at 10 years post-operatively. All patients received a primary ACL reconstruction with direct post-operative visualisation by 3D-CT scan in the University Medical Centre between January 2007 and December 2009. The participants in this study were enrolled in a previous double-blind, randomised controlled trial (RCT). The RCT compared the precision and accuracy of computer-assisted surgery (CAS) with conventional ACL reconstruction [31]. The Medical Ethics Committee of the Erasmus University Medical Centre approved the study protocol of the RCT and the follow-up study (METC-2006–223 and METC-2019–0369) and all participants provided written informed consent.

### Inclusion of patients

Patients were included if they were 18 years or older, were eligible for primary ACL reconstruction and received a post-operative 3D-CT. The occurrence or absence of meniscal injury was registered and treated when necessary. Patients were excluded if they had additional posterior cruciate ligament or collateral ligament injury or if they had insufficient comprehension of Dutch or English. Patients were lost to follow-up if they were unreachable, unable or unwilling to comply with the 10-year post-operative follow-up.

### Surgery

Two fellowship-trained orthopaedic surgeons, who both perform more than 100 ACL reconstructions annually, performed all ACL reconstructions of this study. The ACL reconstruction was performed using an arthroscopic, single-incision, single-bundle, trans-tibial surgical technique, using either bone–patella–tendon–bone (BPTB) or a looped semi-tendinosus, gracillis autograft. The choice for either graft was made individualised per patient by the surgeon pre-operatively depending on specific athletic demands and other knee comorbidities. The graft diameters ranged from 8 to 11 mm. Patients received conventional surgery or CAS, based on randomisation in the previous RCT. CAS was performed using a stand-alone infrared controlled computer (Version 1.0, Brainlab, München, Germany). This system uses per-operatively acquired radiographs of the knee. These are then used to template the femoral and tibial tunnel position in the computer.

The conventional femoral and tibial bone tunnels were positioned within the native anatomic ACL footprint. The aimed position of the tibial tunnel aperture in patients operated with CAS was located at 44% of the anterior-to-posterior length of the tibial plateau between the eminences [39, 47]. The aimed femoral tunnel position with CAS was located at the origin of the anteromedial bundle. In the posterior-to-anterior direction, the aimed femoral aperture position was located at 24.8% measured parallel to the Blumensaat line. In the superior-to-inferior direction, the aimed femoral aperture position was located at 28.5% of the height perpendicular to the Blumensaat line [5].

### Outcome measures

Patients were invited to fill in questionnaires and to visit the outpatient clinics for physical examination pre-operatively, at 2-year and 10-year follow-up, besides their regular post-operative follow-up. The questionnaires could be filled in either online or on paper. Physical examination consisted of manual and arthrometric laxity measurement. Arthrometric laxity measurement was performed using the KT-1000 (Medtronic, Cal. U.S.A.). The same experienced orthopaedic surgeon examined all patients pre-operatively, at 2-year, and 10-year follow-up, blinded for tunnel positions. Ipsilateral graft ruptures, contralateral ACL ruptures and other adverse events were registered for both knees.

There were two primary outcomes in this study: the absolute score of the International Knee Documentation Committee Subjective Knee Form (IKDC-SKF) and the overall failure at 10-year follow-up. The IKDC-SKF, provides a responsive outcome measurement of patients’ perception of symptoms, knee function and ability to participate in sports activities after ACL reconstruction [18, 56]. The IKDC-SKF is a valid tool for patients with ligament or meniscal ruptures with good test–retest reliability, with an interclass correlation between 0.87 and 0.95 [14]. Overall failure was defined if one or more of the following criteria were present: a visualised graft rupture on MRI or revision surgery during follow-up (described as ‘graft failure’), knee laxity during physical examination (Lachman ≥ 2 + , pivot shift ≥ 2 + , KT-1000 difference ≥ 4 mm or an IKDC objective score of C or D) or an IKDC-SKF score < 50. The secondary outcome was radiographic osteoarthritis. Weight-bearing antero-posterior and lateral radiographs of both knees were made at 10-year follow-up. These were graded with the Kellgren and Lawrence (K&L) classification for osteoarthritis by two researchers, blinded for patient allocation [22]. Radiographic osteoarthritis was defined as a K&L score of ≥ grade 2 on the operated knee.

### Measurement of the tunnel aperture positions

The CT scan of the operated knee was made within 72 h post-operatively in all patients. A 64 channel multi-slice technology CT-scanner (Somatom, Siemens Medical Solutions, Forchheim, Germany) with helical acquisition in 1.0 mm sections (120 kV, 160 mAs, rotation time 1.0 s) was used to determine the tunnel positions. The knee CT imaging was performed from the top of the suprapatellar pouch to the superior tibial and fibula diaphysis, post-operatively. The data were then transferred and blinded for patient, into the 3-dimensional measurement software. (MeVisLab Version 2.0, MeVis Medical solutions AG, Bremen, Germany).

**Fig. 1:**
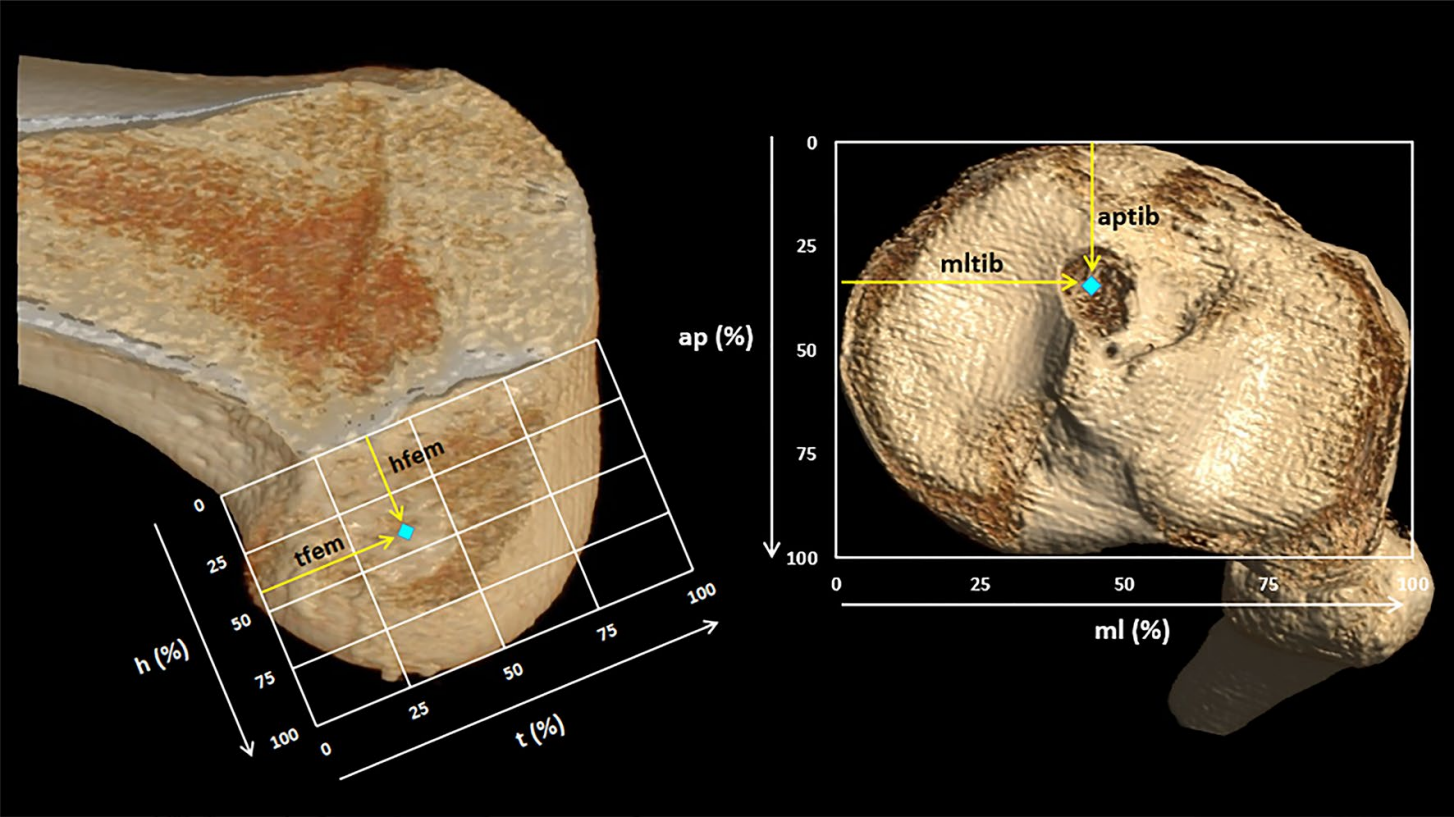
3D-CT view of the medial side of the lateral femoral condyle with the quadrants of Bernard (left) and the tibial plateau (right). Blue dot: Example tunnel aperture position. Femur: h: the total superior-inferior height of the quadrants, starting at 0% perpendicular at the Blumensaat line and ending at 100% at the end of the femoral condyle. t: the total posterior-to-anterior length of the quadrants, starting at 0% at the most posterior part of the condyle, parallel to Blumensaat and ending at 100% at the most anterior part. tfem: posterior-to-inferior position of the tunnel aperture, expressed in a percentage of t. hfem: superior-inferior position of the tunnel aperture, expressed in a percentage of h. Tibia: ap: the total anterior-to-posterior length of the quadrants, starting at 0% at the most anterior part of the tibial plateau and ending at 100% at the most posterior part of the tibial plateau. ml: the total medial-to-lateral length of the quadrants, starting at 0% at the most medial part of the tibial plateau and ending at 100% at the most lateral part of the tibial plateau. aptib: anterior-to-posterior position of the tunnel aperture, expressed in a percentage of ap. mltib: medial-to-lateral position of the tunnel aperture, expressed in a percentage of ml

Measurement of the three-dimensional images was performed by a radiologist blinded for patient allocation. The anatomic coordinate axis method was used for measurement [25, 30, 52]. Using the three-dimensional tri-axial properties of the desktop version of MeVis, the contour of the femur (the intra-condylar axis and medial side of the lateral femoral condyle) and tibia (circumference of the tibial plateau) was mapped. The apertures of the femoral and tibial tunnel were mapped using the centre of the tunnel opening. All these measurements were performed and translated into a true sagittal view of the femoral condyle and a true transversal view of the tibial plateau. The femoral and tibial tunnel aperture positions were then translated into percentages of respectively the femoral condyle and the tibial plateau (Fig. 1).

### Statistical analysis

Statistical analysis was performed with the use of SPSS software (IBM SPSS Statistics for Windows, Version 25.0. Armonk, NY: IBM Corp.). The Shapiro–Wilk analysis was performed to test for normal distribution of variables. Paired *t* tests were executed to test the changes in patient-reported outcome between baseline and 10-year follow-up. The intra-class correlation coefficients (ICC) were calculated to determine inter-observer reliability for tunnel position measurement. Linear regression analyses were performed to determine the relation between IKDC-SKF score (dependent variable) and the different tunnel aperture positions (independent variables). Logistic regression analyses were performed to determine the relation between the dichotomous dependent variables (overall failure and osteoarthritis) and tunnel aperture positions (independent variables). There was adjusted for the following variables: sex, Body Mass Index (BMI), type of surgery (computer-assisted or conventional), type of graft (bone-patella-tendon-bone or hamstring), meniscal tears, chondral defects (both observed per-operatively) and pre-operative Lachman score. There was also specifically adjusted for pre-operative IKDC-SKF and pre-operative grade of osteoarthritis in the analysis of respectively IKDC-SKF and osteoarthritis. In order to identify the safe zone for tunnel aperture positions, we performed the Youden’s index in conjunction with receiver operating characteristic (ROC) analysis. A *p* value less than 0.05 was considered statistically significant. *P* values, 95% confidence intervals (95% CI) and odds-ratios were reported with two decimals, all other values were reported with one decimal in tables and in text. No sample size calculation was performed since there was a fixed study population because it was the follow-up of a previous RCT.

## Results

### Study population and baseline characteristics

**Fig. 2:**
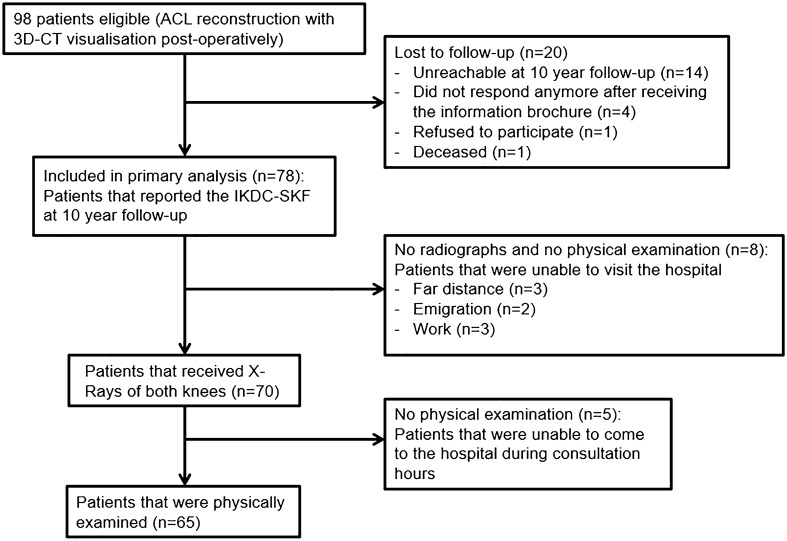
Flow chart of the patients in the study

Of the 98 patients that received primary ACL reconstruction with direct 3D-CT visualisation, 78 patients reported the IKDC-SKF at 10-year follow-up and were included in the primary analysis. A flowchart of the patients in the study can be seen in Fig. 2.

The mean follow-up duration was 11.4 years and ranged from 9.7 years until 12.7 years. The baseline and per-operative characteristics are presented in Table 1. There were no significant differences in baseline and per-operative characteristics between the included and the excluded cases.

**Table 1:** Baseline and per-operative characteristics of patients (*n* = 98)

	Included (*n* = 78)	Lost to follow-up (*n* = 20)	*P* value
Age at operation, years	24.9 (21.0–30.1)	27.0 (21.6–31.5)	(n.s.)
Gender, *n* (%)			(n.s.)
Male	58 (74.4)	17 (85.0)	
BMI at operation, kg/m^2^	23.9 (22.7–25.6)	25.2 (23.7–27.1)	(n.s.)
Graft type, *n* (%)			(n.s.)
BPTB	33 (42.3)	13 (65.0)	
Hamstring	45 (57.7)	7 (35.0)	
Type of surgery, *n* (%)			(n.s.)
CAS	36 (46.2)	9 (45.0)	
Conventional	42 (53.8)	11 (55.0)	
Tunnel aperture positions			
tfem	39.1 (9.4)	40.4 (9.6)	(n.s.)
hfem	38.5 (9.0)	38.7 (9.9)	(n.s.)
aptib	38.8 (6.3)	37.8 (6.4)	(n.s.)
mltib	42.4 (4.8)	44.0 (4.7)	(n.s.)
Meniscal tear per-operative, *n* (%)			(n.s.)
No tear	55 (70.5)	15 (75.0)	
Medial tear	7 (9.0)	1 (5.0)	
Lateral tear	15 (19.2)	4 (20.0)	
Combined	1 (1.3)	0 (0.0)	
Chondral defect per-operative, *n* (%)			(n.s.)
No defect	53 (67.9)	14 (70.0)	
Patellar	2 (2.6)	1 (5.0)	
Medial	12 (15.4)	3 (15.0)	
Lateral	4 (5.1)	1 (5.0)	
Combined	7 (9.0)	1 (5.0)	

*tfem* posterior-to-anterior femoral tunnel position, *hfem* superior-inferior femoral tunnel position, *aptib* anterior-to-posterior tibial tunnel position, *mltib* medial-to-lateral tibial tunnel position

Median value and (interquartile range) is presented for age and BMI

Mean value (standard deviation) is presented for the tunnel aperture positions

Frequency (percentage) is presented for the other characteristics

**Fig. 3:**
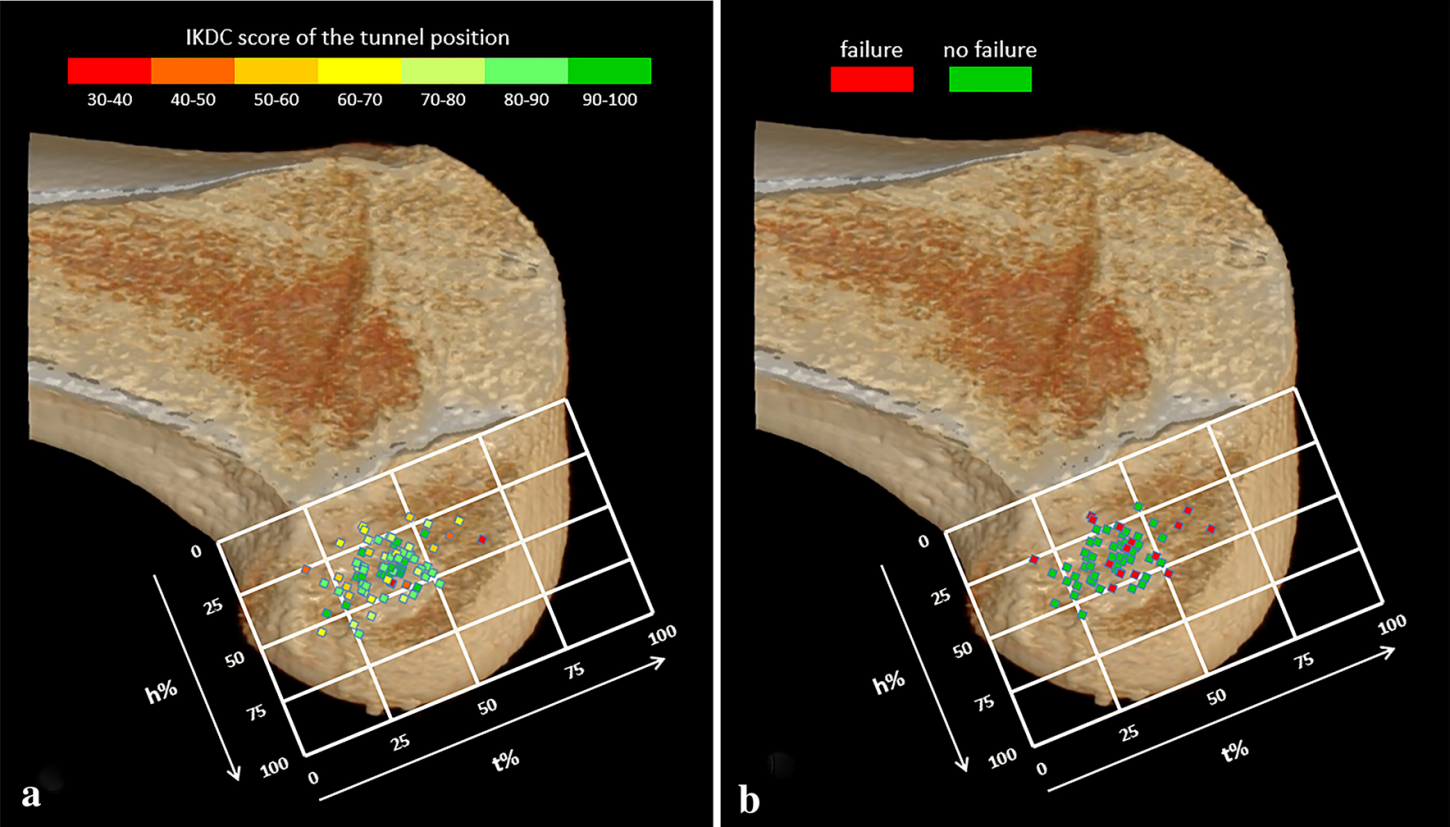
Tunnel aperture positions in the femur. **A** displays the IKDC-SKF score of each aperture position. **B** displays per aperture position if overall failure was present or not present

**Fig. 4:**
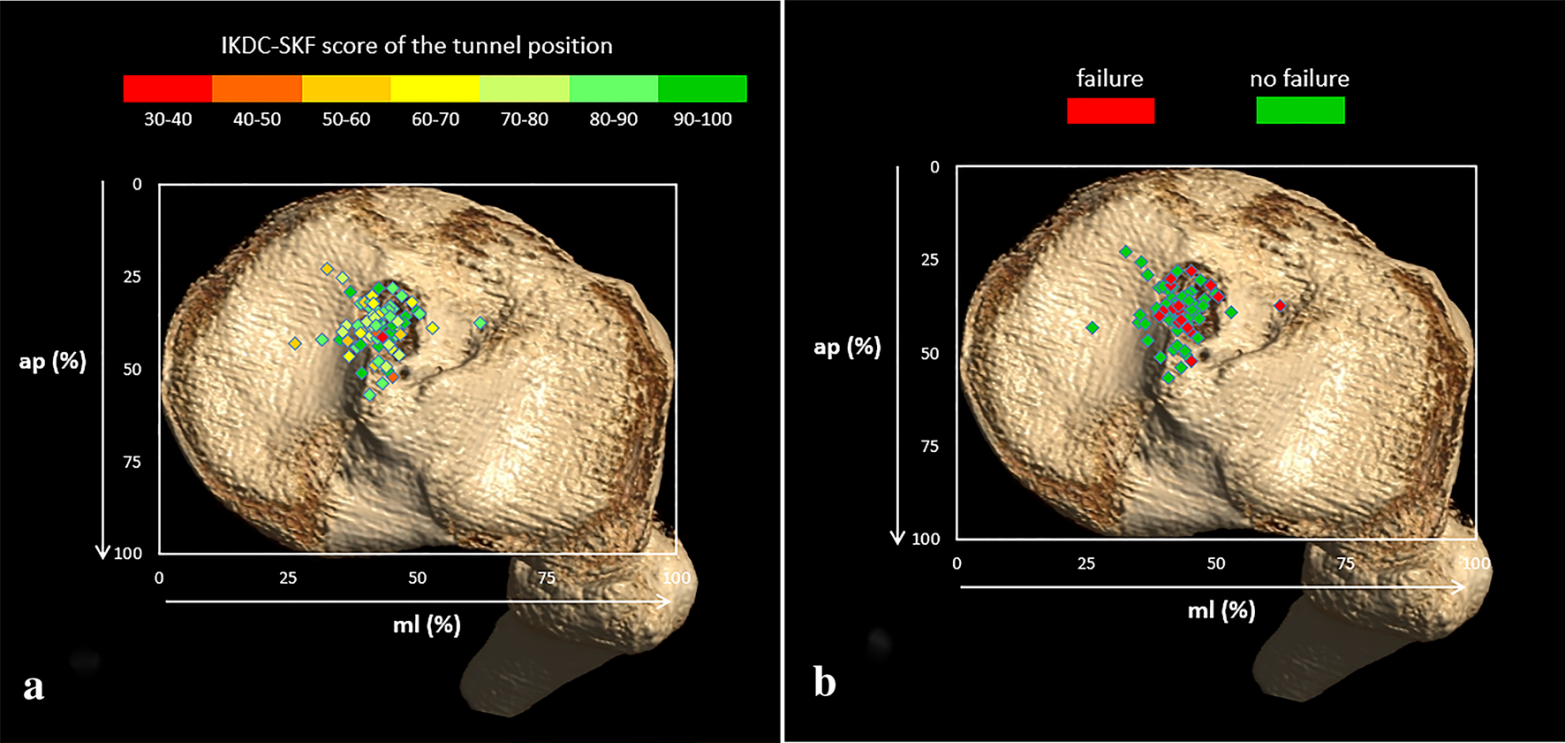
Tunnel aperture positions in the tibia. **A** displays the IKDC-SKF score of each aperture position. **B** displays per aperture position if overall failure was present or not present

The femoral tunnel aperture positions ranged between 16.7 and 65.3% of the posterior-to-anterior distance and between 17.5 and 60.0% of the superior-inferior height. The tibial tunnel apertures ranged between 22.9 and 56.9% of the anterior-to-posterior distance and between 26.1 and 62.1% of the medial-to-lateral distance (Figs. 3A, 4A).

Inter-observer measurement of the three-dimensional tunnel aperture positions showed an ICC of the femoral positions of 0.90 (confidence interval (CI) 0.85–0.93) and of the tibial positions was 0.99 (CI 0.98–0.99).

Median value and (interquartile range) is presented for age and BMI. Mean value (standard deviation) is presented for the tunnel aperture positions. Frequency (percentage) is presented for the other characteristics. tfem = posterior-to-anterior femoral tunnel position, hfem = superior-inferior femoral tunnel position, aptib = anterior-to-posterior tibial tunnel position, mltib = medial-to-lateral tibial tunnel position.

### Primary outcome

No significant associations were found between the tunnel aperture positions and the IKDC-SKF score at 10-year follow-up (Figs. 3A, 4A and Table 2). Additionally, also no significant associations between the tunnel aperture positions and the IKDC-SKF score at 2-year follow-up were found.

**Table 2:** Relations between tunnel aperture positions and primary outcomes

	tfem	hfem	aptib	mltib
*IKDC-SKF n = 78*				
B Coefficient	0.05	0.20	0.14	0.50
95% CI	(− 0.38; 0.48)	(− 0.26; 0.67)	(− 0.56; 0.84)	(− 0.35; 1.32)
*p* value	(n.s.)	(n.s.)	(n.s.)	(n.s.)
*Overall failure n = 65*				
Odds ratio	1.09	0.98	0.91	1.17
95% CI	(1.01; 1.18)	(0.91; 1.06)	(0.80; 1.04)	(0.99; 1.39)
*p* value	0.03	(n.s.)	(n.s.)	(n.s.)
*Osteoarthritis* *n* = 70				
Odds ratio	0.99	0.99	0.89	0.99
95% CI	(0.92; 1.06)	(0.92; 1.07)	(0.78; 1.01)	(0.87; 1.14)
*p* value	(n.s.)	(n.s.)	(n.s.)	(n.s.)

*tfem* posterior-to-anterior femoral tunnel position, *hfem* superior-inferior femoral tunnel position, *aptib* anterior-to-posterior tibial tunnel position, *mltib* medial-to-lateral tibial tunnel position

The outcome variables presented are the IKDC-SKF, overall failure and osteoarthritis *(italics)*

*N* describes the number of participants that were analysed per outcome variable

Adjusted values are presented

The IKDC-SKF showed significant improvement between the pre-operative measurement and 10-year follow-up. The mean IKDC-SKF score increased from 63.7 (± 14.2) pre-operatively to 77.5 (± 16.1) at 10-year follow-up. This increase of 13.8 points was statistically significant (*p* = 0.00) and exceeded the minimum clinically important difference of 11.5 points [18]*.* At 2-year follow-up, the IKDC-SKF score was highest, with a mean score of 85.8 (± 13.3). At 10-year follow-up, 11 patients (14.1%) had a graft failure and 9 patients had a contralateral ACL failure (11.5%).

At 10-year follow-up, 16 patients (24.6%) were classified into the overall failure group (Table 3). In this group, 6 patients had BPTB grafts and 10 patients had hamstring grafts. The femoral posterior-to-anterior tunnel aperture position (tfem, *p* = 0.03) showed a significant positive relation with overall failure. The mean posterior-to-anterior femoral tunnel aperture position (tfem) of the ‘no failure’ group was 37.7% and was 44.1% in the ‘failure’ group. Youden’s index indicated that the cut-off point was at 35.0%. Femoral tunnel apertures placed further anteriorly than 35% in posterior-to-anterior direction showed increased overall failure. Of the 16 overall failures, 15 (93.8%) were placed further anteriorly than the cut-off point of 35%. The femoral height positions and tibial tunnel aperture positions did not show significant relations with overall failure (Figs. 3B, 4B and Table 2).

**Table 3:** Criteria for overall failure

Criterion	Number of cases
Graft failure	3 (19%)
Knee laxity during physical examination	5 (31%)
IKDC-SKF < 50	4 (25%)
Multiple criteria (≥ 2 criteria)	4 (25%)
Total overall failure	16 (100%)

Frequencies (percentages) are presented

### Secondary outcome

We found no significant associations between the tunnel aperture positions and the development of osteoarthritis (Table 2). Twenty-two patients (28.2%) developed mild to severe osteoarthritis (K&L grade 2 to 4) in the operated knee.

## Discussion

This study shows that the femoral tunnel aperture position did significantly affect overall failure. The tibial tunnel aperture position did not significantly affect overall failure. The femoral and tibial tunnel aperture positions did not significantly affect the IKDC-SKF and the development of osteoarthritis at long-term follow-up. The overall patient-reported and clinical outcome of ACL reconstruction was good to excellent.

The results of this study according to the IKDC-SKF are most comparable with the long-term follow-up study of Sundemo et al. In their follow-up of 16 years, no correlation between the femoral tunnel position and the IKDC-SKF score was found either [50]. However, these long-term findings are in contrast with the short-term findings of other studies that demonstrate that anatomic tunnel positions show superior results over non-anatomic positions. These demonstrated that posteriorly placed femoral tunnels, ranging between 25 and 40% of the posterior-to-anterior distance along Blumensaat, had positive effect on patient-reported outcome [6, 21, 36, 43, 46]. Tibial tunnels ranging between 32 and 46% of the anterior-to-posterior distance of the plateau influenced patient-reported outcome positively [4, 36, 43]. A possible explanation for the absent relation between the reported outcome scores and the tunnel positions is the subjectivity of patient-reported outcome scores. Therefore, these scores are sensitive for interpretation bias. As could be observed in the questionnaires, some patients who reported low IKDC scores were paradoxically still able to participate in high-level or high-intensity sports. Vice versa, patients that have proven clinical graft laxity can still report excellent subjective outcome [12].

The femoral tunnel aperture positions did significantly affect the other primary outcome, overall failure. Femoral tunnel apertures placed further posteriorly in the femoral condyle showed a decrease in overall failure at long-term follow-up. This signifies the importance of posterior placement of the femoral tunnel apertures to resemble anatomy and to reduce failure rates. The cut-off point for this posterior zone was identified at 35.0%. No significant association was found between femoral superior–anterior height and tunnel aperture position. This study therefore identifies the posterior 35% in posterior-to-anterior direction as a safe zone for femoral tunnel aperture placement. However, the surgeon should always take the risk of a posterior wall blowout into account when placing the femoral tunnel too close to the posterior articular margin [34]. Also, different studies do indicate the anatomical insertion of the ACL lies just above the midline in superior-inferior height [28, 60].

The decrease in overall failure is due to the anatomic resemblance of the original ACL of posteriorly placed femoral tunnels. Overall, anterior femoral tunnel positions lead to more vertically oriented grafts that allow more antero-posterior and rotational translation. This results in more rotational instability and increases chondral and meniscal stress [1, 28, 41, 53, 59]. Overall, anterior femoral tunnel positions can also lead to graft impingement in the intercondylar roof [28, 54].

The findings on overall failure correspond with the short-term findings of several studies. These studies reported that anatomical, more oblique graft positions gave superior clinical outcome, increased rotational stability and lower revision rates [3, 7, 21, 24, 43, 45, 53]. These findings also correspond with the long-term findings of Pinczewski et al., who state that more vertical inclination is associated with increased rotational instability [41]. However, the previously mentioned study of Sundemo et al. did not show a correlation with the femoral tunnel position and clinical outcome (e.g. Lachman, pivot shift test and KT-1000) [50]. This can be explained because they retrospectively assessed the tunnel positions on radiographs. Therefore, they had to exclude graft ruptures and contralateral ruptures from their study. In this study, the tunnel positions were determined directly post-operative and ruptures were included in the overall failure group.

The tibial tunnel aperture positions did not affect overall failure, which is contradictive to the findings of Inderhaug et al. [15]. Their study suggests that posteriorly placed tunnels (> 50% of anterior-to-posterior distance) show increased rotational instability. The absence of a tibial relation with overall failure could be explained by the relatively small range tibial anterior-to-posterior tunnel aperture positions. The tibial aperture positions in our study ranged until 56.9% of the anterior-to-posterior distance with a mean of 38.8%, whilst Inderhaug et al. only called placement too posteriorly if it was over 50%. Inderhaug et al. also assessed the tunnel positions on radiographs, which offers a less precise visualisation compared to 3D-CT [25, 30]. However, this study did find a non-significant trend on overall failure according medial-to-lateral tunnel positions. This trend indicates that more laterally placed tunnels show increased overall failure. This trend can again be explained by the instability and inferior results associated with more vertically oriented grafts [24, 43, 53].

Osteoarthritis did not show a significant relation with tunnel aperture positions. This corresponds with the findings of the long-term study from Sundemo et al. [50]. The prevalence of osteoarthritis (31.0%) was lower compared to other long-term follow-up [37, 49]. The prevalence of graft failures was higher than the prevalence described in the literature [27]. This can be caused by our definition of graft failures, which also includes partially ruptured grafts and need for revision surgery.

In general, ACL reconstruction provided favourable increases of both short- and long-term patient-reported outcome that were significant and clinically relevant, similar to other cohorts [13, 44]. The decrease in IKDC-SKF between 2- and 10-year follow-up can be caused by multiple factors, such as lower activity levels because of the negative effect of ageing and the occurrence of comorbidities [29].

3D-CT was used to assess the tunnel positions directly post-operative. 3D-CT is the most accurate measurement tool with the highest inter-observer ICC (> 0.993) and intra-observer ICC (> 0.963). Therefore, the established anatomic coordinate axis method 3D-CT provides the most reliable representation of the anatomy [25, 30, 52]. This is particularly important in visualising the femoral condyle with its convex shape. This was the first study that coupled directly visualised tunnel positions by 3D-CT to long-term outcome. Besides that, this study was amongst the first studies that coupled tunnel positioning in general to long-term outcome.

There were limitations present in this study. All patients in this study were operated with the trans-tibial technique. Several studies have shown that the anteromedial technique achieves a more anatomic tunnel placement, improved antero-posterior and rotational knee stability [10, 26, 33, 38, 42, 48, 51, 58]. However, there is inconsistency between these different studies if the improved graft anatomy of the anteromedial technique leads to better reported and clinical outcome. Although there was adjusted for possible confounders, there was not adjusted for patient-specific morphologic knee differences. Because generalised figures of 3D-CT were used, some tunnel apertures appear to be positioned outside of the anatomic boundaries (e.g. in the medial tibial plateau) (Figs. 3 and 4). There were indeed outliers in tunnel positions, but these were still positioned within the anatomic boundaries of the individuals’ tibia and femur. The outliers were mostly due to anatomical variations [16, 19, 35]. This study analysed both the femoral tunnel position and tibial tunnel position as two independent parameters but it is clear that these positions influence each other. The choice of independent assessment was based on multiple studies that also assessed the tunnel positions independently [3, 4, 9, 15, 16, 20, 21, 36, 50]. The number of patients that were physically examined was smaller because some patients were unable to come to the hospital. Therefore, the overall failure and osteoarthritis group had a reduced sample size, the IKDC-SKF score did not have a reduced sample size. Since there was a fixed number of study participants because it was a follow-up, there was no power analysis performed to determine the sample size. This was an exploratory study that researched the long-term effects of a relative spread of tunnel positions. The results of this study offer guidance to surgeons to operate more precisely and accurately and reconstruct a long-lasting graft. Future research that compares the effect that anatomical versus non-anatomical tunnel positions have on long-term outcome is required to confirm the findings of this study.

## Conclusion

Femoral and tibial tunnel positions were not associated with long-term patient-reported outcome and radiographic osteoarthritis. Long-term overall failure was more frequently seen in patients with a more anteriorly placed femoral tunnel. This study identified a safe zone located at the most posterior 35% of the femoral condyle parallel to Blumensaat.
